# Microlearning Reduces Unnecessary Antibiotic Provision for Open Hand Injuries

**DOI:** 10.7759/cureus.74910

**Published:** 2024-12-01

**Authors:** Edward J Armstrong, Aiman Jamal, Christine Quinlan

**Affiliations:** 1 Urology, Royal Berkshire NHS Foundation Trust, Reading, GBR; 2 Ophthalmology, University Hospitals Dorset NHS Foundation Trust, Dorset, GBR; 3 Plastic and Reconstructive Surgery, Mater Misericordiae University Hospital, Dublin, IRL

**Keywords:** antibiotic stewardship, hand injuries, microlearning, quality improvement, trauma

## Abstract

Background

Antimicrobial stewardship approaches are important in managing the growing global challenge of antibiotic resistance. Microlearning describes educational interventions involving small, digestible chunks of information focussed on a single learning objective.

Aim

This quality improvement project aimed to improve the compliance of antibiotic administration for open hand injury referrals with national and local guidelines using a microlearning educational intervention.

Method

Antibiotic administration for open hand injury referrals was audited at baseline, following the creation and dissemination of an infographic displaying the indications for antibiotics, and again after the creation and dissemination of a short video providing the same information.

Result

The proportion of referrals with no indication for antibiotics that were given or recommended antimicrobials fell from 50.0% (22/44) to 14.3% (5/35) after dissemination of the infographic and short video.

Conclusion

Microlearning educational interventions are effective at improving compliance with clinical guidelines in this context.

## Introduction

Antimicrobial resistance is a growing global challenge, with 4.95 million deaths worldwide and 35, 200 deaths in the UK associated with drug-resistant infections in 2019 [1]. Antimicrobial stewardship is an 'organizational or healthcare system-wide approach to promoting and monitoring judicious use of antimicrobials to preserve their future effectiveness’ [2]. This approach involves a combination of interventions including monitoring and evaluating antimicrobial prescribing, providing regular feedback to antimicrobial prescribers, and providing education and training for healthcare practitioners [3].

Open hand trauma is a common presenting injury to emergency departments (EDs), accounting for 2.6 million presentations in the US every year [4]. National UK guidelines for antibiotic use in open hand injuries, developed by the British Society for Surgery of the Hand (BSSH), are available to all clinicians via their Hand Trauma App [5]. These guidelines recommend prophylactic antibiotics in the following situations: animal or human bite wounds, amputations, injuries with associated fractures or joint abnormalities, and significant wound infection or contamination. Local guidelines at Oxford University Hospitals (OUH) recommend antibiotics in the same situations but specify they are not required for open tuft fractures. In addition, large soft tissue injuries over 5 cm in length, with devitalized tissue present, or in patients who are diabetic require antibiotic prophylaxis [6].

The definition of microlearning is not universal [7], however, most descriptions recognize microlearning involves small, digestible chunks of information focussed on a single learning objective, available on demand, and usually delivered via digital technology [8, 9]. Microlearning is a growing educational trend with successful proliferation in computer and medical sciences [8]. One educational theory underpinning the use of microlearning in healthcare contexts is ‘just-in-time training’ (JITT) which refers to the provision of tailor-made, immediate, and focused learning resources when they are needed most; for healthcare students and providers, at the point of care with a patient [10].

A 2019 scoping review found that microlearning aimed at health professions students can facilitate flexible and accessible learning with the potential to improve performance and increase patient safety [7]. Some previous work has highlighted the application of microlearning for teaching and upskilling postgraduate doctors, recognizing traditional didactic lectures are often difficult to facilitate for physicians-in-training who have competing clinical responsibilities and growing administrative tasks [11, 12]. Despite the intuitive potential for microlearning in postgraduate medical education, relatively few studies have been published reporting or discussing microlearning initiatives in this group [11-14].

This quality improvement project aimed to improve the compliance of antibiotic administration for open hand injury referrals with local guidelines in a plastic surgery unit at a United Kingdom (UK) university hospital using a microlearning educational intervention.

## Materials and methods

Clinical context

OUH is one of the largest National Health Service (NHS) teaching trusts in the UK, comprising four main hospitals with 1036 general and acute care beds and 58 adult critical care beds [15].

Acute referrals to the OUH plastic surgery team are documented on the online acute referral system (OARS), an electronic system developed to manage the complex multi-person and multi-team discussions that take place when seeking specialist advice [6]. Referrers can refer patients directly on OARS or make a telephone referral to an on-call resident doctor, who documents the referral discussion on OARS. Referrers include the OUH emergency departments (ED), general practitioners (GP), regional minor injuries units (MIU), and clinicians at other NHS trusts seeking specialist advice.

Electronic OARS referrals are completed by answering several branching algorithmic questions that provide clinical advice in line with local guidelines. There is also a free text box where referrers can document other information along with any additional steps they have taken, and plastic surgery team members can record any advice provided over the telephone and reply to referrers directly via OARS.

Plan-do-study-act (PDSA) methodology

The PDSA method originates from industry [16] and describes a four-stage cyclic learning approach to adopting and adapting changes aimed at quality improvement. The ‘plan’ stage involves identifying a change aimed at improvement, the ‘do’ stage implementing the change, the ‘study’ stage assessing the impact of the change, and the ‘act’ stage adapting the change and formulating the next steps [17]. We aimed to introduce small-scale, pragmatic, and iterative change, and so adopted the PDSA methodology for this project [18].

Baseline data collection

We reviewed all OARS referrals from two non-continuous weeks between 23/3/22-30/3/22 (inclusive) and 6/4/22-13/4/22. This period was chosen to straddle the rotation of resident doctors and capture advice given by one resident doctor cohort at the end of their rotation in the department and another at the beginning.

Data was collected for referrals involving open hand injuries and extracted by reviewing the OARS electronic referral documentation along with any clinical pictures submitted by the referrer. The parameters recorded for each referral are summarised in Table 1.

**Table 1:** Parameters recorded for each referral

Referral parameter
Referrer details:
Referral centre
Electronic documentation completed by referrer or plastic surgery team member
Injury details:
Description of the mechanism of injury
Description of injury
Indications for antibiotics:
Amputation
Bite wound
Open fracture or dislocation (excluding tuft fracture)
Open joint or deep laceration over joint
Significant wound infection or contamination
Laceration > 5 cm
Devitalized tissue present
Patient with diabetes
Antibiotics advised or given:
Antibiotics given by referrer in free text
Antibiotics advised by OARS algorithm
Antibiotics advised by plastic surgery team member in free text

Any uncertainty regarding indications for antibiotics was resolved through a discussion between the authors (EA and AJ, resident doctors). Any disagreement was resolved through discussion with the third author (CQ, consultant plastic surgeon).

PDSA cycle 1

The change we identified in the ‘plan’ stage was an educational intervention to improve resident doctors' understanding of the indications for antibiotics in the context of open hand injuries.

**Figure 1:**
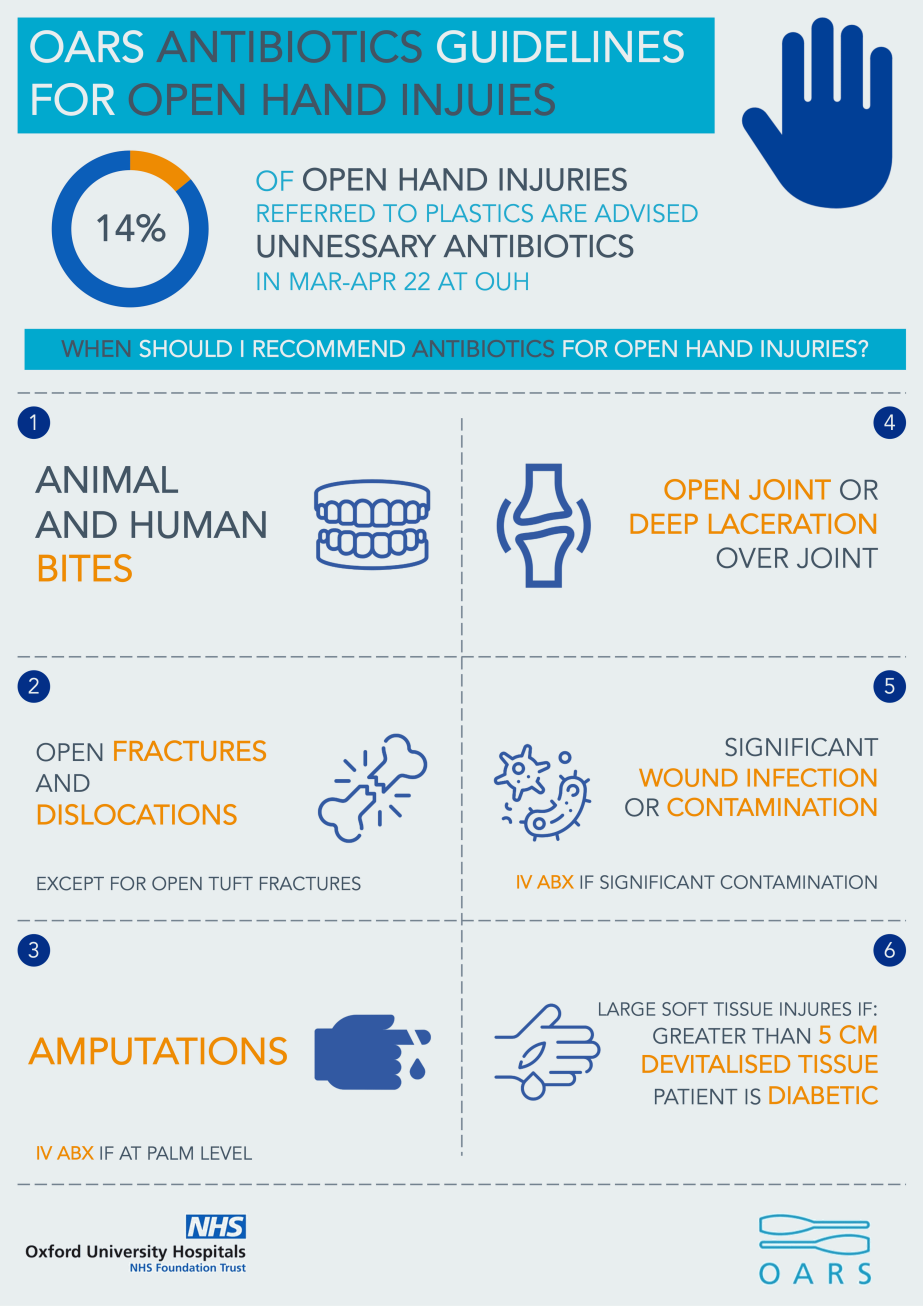
Infographic used in PDSA cycle 1 This figure was created by the authors as part of this project.

We designed an infographic poster that included a summary of the baseline data collected and a simplified representation of the relevant indications with accompanying pictograms (Figure 1). The color scheme was chosen to conform to the NHS Identity Guidelines [19]. The infographic was printed and posted around the Plastic Surgery doctors’ office and department and emailed to all resident doctors involved in plastic surgery referrals.

Data collection was repeated over one week 25/7/22-31/7/22 (inclusive) using the methodology described above. A one-week period was chosen to expedite data collection and permit rapid iteration of the next cycle.

PDSA cycle 2

We adapted the educational intervention from PDSA cycle 1 into a short video including the same information as the infographic poster. The video was made on Animaker, an artificial intelligence (AI)-powered platform accessible to non-designers [20]. The video was created in a 450 px x 450 px resolution to facilitate download and viewing on mobile devices and laptops. The video was shared on the departmental Whatsapp group and emailed to all resident doctors involved in Plastic Surgery referrals.

Data collection was repeated over two weeks 18/10/24-01/11/24 (inclusive) using the methodology described above.

Data analysis

Data was analyzed using Microsoft Excel and R Studios Team (Integrated Development for R. RStudio, PBC, 2020, Boston, MA). Pearson’s Chi-squared test was used to establish statistical significance. Fisher’s Exact test was used where Chi-squared assumptions could not be met.

## Results

Baseline data collection

A hundred and twenty-eight referrals for open hand injuries were made during the two-week period (Table 2). OUH emergency departments made the most referrals (46.9%, 60) and electronic documentation was completed by Plastic Surgery team members in 50.0% (64) of cases.

**Table 2:** Summary of referral source by cohort Data represented as frequency (%). Data was analysed using Fisher’s Exact test and Pearson’s Chi-squared test. p < 0.05 is considered statistically significant. PDSA: plan-do-study-act; ED: emergency department; MIU: minor injuries unit; GP: general practice; NHS: National Health Service.

Characteristic	Baseline data (frequency (%))	PDSA cycle 1 (frequency (%))	PDSA cycle 2 (frequency (%))	Test and statistic	p value
Number of patients	128	52	90	N/A	N/A
Referral source:				Fisher’s Exact test	0.664
ED	60 (46.9)	25 (48.1)	47 (52.2)		
MIU	36 (28.1)	13 (25)	21 (23.3)		
GP	4 (3.1)	1 (1.9)	6 (6.7)		
Other NHS hospitals	25 (19.5)	13 (25)	16 (17.8)		
Other	3 (2.3)	0 (0)	0 (0)		
Electronic referral documentation completed by:				C^2^ = 1.07	0.585
Plastic surgery team member	64 (50.0)	25 (48.1)	49 (54.4)		
External referral	64 (50.0)	27 (51.9)	41 (45.6)		

At least one indication to give antibiotics was present in 65.6% (84) of referrals (Table 3).

**Table 3:** Primary indications for antibiotics by cohort Data represented as frequency (%). Data was analyzed using Pearson’s Chi-squared test. p < 0.05 is considered statistically significant. PDSA: plan-do-study-act.

Indication to give antibiotics present	Baseline data (frequency (%))	PDSA cycle 1 (frequency (%))	PDSA cycle 2 (frequency (%))	Test and statistic	p value
Injuries with an indication to give antibiotics	84 (65.6)	36 (69.2)	55 (61.1)	Fisher’s Exact test	0.872
Injuries without an indication to give antibiotics	44 (34.4)	16 (30.1)	35 (38.9)		

**Figure 2:**
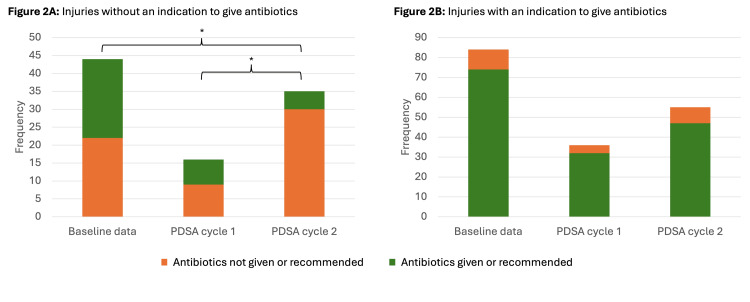
Injuries given antibiotics prior to referral or recommended antibiotics Data is represented as frequency. Data was analyzed using Fisher’s Exact test and Pearson’s Chi-squared test. p < 0.05 is considered statistically significant. *indicates statistical significance. PDSA: plan-do-study-act.

Of the 44 (34.4%) injuries without an indication to give antibiotics, 22 (50%) were unnecessarily advised antibiotics or given antibiotics before referral (Figure 2A). About 14.1% (18) of all injuries referred were advised unnecessary antibiotics. Of the 84 (65.6%) injuries with an indication to give antibiotics, 10 (12%) were neither given nor recommended antibiotics (Figure 2B).

PDSA cycle 1

Fifty-two referrals were reviewed during the one-week period. There was no statistically significant difference in the referral source profile (Table 2), or the proportion of injuries with an indication to give antibiotics (Table 3) between any cohort.

Of the sixteen (30.1%) referrals without an indication to give antibiotics, seven (43.8%) were unnecessarily given or advised antibiotics (43.8%) (Figure 2A). This represented a small decrease from the baseline cohort that was not statistically significant.

There was no statistically significant difference in the proportion of injuries with an indication to give antibiotics that were given or recommended antimicrobials between any of the three cohorts. (Figure 2B)

PDSA cycle 2

Ninety referrals were reviewed during the two-week period (Table 2). Of 35 (38.9%) referrals without an indication to give antibiotics, five (14.3%) were given or recommended antibiotics, a statistically significant decrease from the baseline cohort (p = 0.002) and PDSA cycle 1 (p = 0.03) (Figure 2A). Of these five referrals, three were given antibiotics before referral by the referrer and two were unnecessarily advised antibiotics on referral.

## Discussion

We report a quality improvement project utilizing PDSA methodology to improve the compliance of antibiotic recommendations for open hand injuries with clinical guidelines. Over two PDSA cycles, the proportion of open hand injuries without an indication for antibiotics given or recommended antimicrobials fell from 50.0% (22/44) to 14.3% (5/35) (p = 0.002). The change implemented was an educational intervention including a short video utilizing the principles of microlearning.

A limited number of other publications also highlight microlearning as an effective pedagogical method for improving awareness and compliance with clinical guidelines in the healthcare environment and even fewer in surgical departments. One other study described the use of short (15-minute) teaching sessions alongside experiential learning to improve the performance of geriatric assessment processes amongst surgical residents, including documentation of medications, functional status, and mobility status at admission [12]. Clinical guidelines are often amenable to breakdown into self-contained segments with specific outcomes so microlearning seems an intuitively useful methodology for the dissemination of this material. The brevity of microlearning content is particularly appropriate for a busy surgical department, where competing clinical workstreams, including clinics, inpatient care, and operating lists can limit the time available for undisturbed formal didactic teaching. The asynchronous approach adopted in this report, specifically our short video compatible with mobile devices, circumvents the need to assemble clinical staff for synchronous teaching and ensures the material is available for revisiting just in time, providing learners with full control over their learning [8].

Co-creation refers to active learner involvement in the design and development of education [21] and is applicable to microlearning where microcontent is often cocreated by end users, contrasting against traditional knowledge transfer between a subject matter expert and learner [22]. The educational intervention in this report was co-created by authors of different grades including resident doctors who were also the target of the intervention. This demonstrates the potential for microlearning as a peer-to-peer teaching methodology among postgraduate doctors. We provided material in different formats (infographic and video), with consistent branding and pictography. This follows recommendations made previously by Nissen et al to provide microlearning content in multiple formats along with spaced opportunities for learning [13]. Our results suggest that the video and infographic in combination was a more effective intervention than the infographic alone.

The educational interventions described in this report were aimed at resident doctors in the plastic surgery department with a clinical role of providing initial advice, including advice on antibiotic prescription, to referrers. As found in the second PDSA cycle, three out of five patients unnecessarily given or recommended antibiotics were prescribed these before referral. Further PDSA iterations, therefore, could expand our microlearning educational intervention to frequent referrers to plastic surgery including the OUH ED and MIUs associated with OUH.

This study evaluated changes to learners’ behavior, specifically changes to advice given to referrers, and therefore the results of this intervention. This corresponds to levels 3 (behavior) and 4 (results) in the Kirkpatrick model of evaluation for training programs [23]. This intervention had a substantial positive impact on patient care by promoting local antibiotic stewardship practice. The salient limitation of this report is the lack of evaluation of learners’ reactions (level 1) and learning (level 2) following our educational intervention; this could be an appropriate focus for further PDSA cycles of the project. PDSA cycle 1 was specifically limited by a smaller sample size studied over a one-week period, likely less reflective of ongoing department practice than the two-week periods studied during baseline and PDSA cycle 2 data collection.

## Conclusions

Antibiotics are frequently recommended unnecessarily for open hand injuries. This quality improvement project demonstrates microlearning can be an effective strategy for the dissemination of clinical guidelines to postgraduate doctors. Microcontent may be particularly effective when co-created and presented in multiple different formats.
